# Persistent detection of Zika virus RNA in semen for six months after symptom onset in a traveller returning from Haiti to Italy, February 2016

**DOI:** 10.2807/1560-7917.ES.2016.21.32.30314

**Published:** 2016-08-11

**Authors:** Emanuele Nicastri, Concetta Castilletti, Giuseppina Liuzzi, Marco Iannetta, Maria R Capobianchi, Giuseppe Ippolito

**Affiliations:** 1 National Institute for Infectious Diseases ‘Lazzaro Spallanzani’, IRCCS, Rome, Italy

**Keywords:** Zika Virus, ZIKV, semen, urine, saliva

## Abstract

A man in his early 30s reported in January 2016 a history of fever, asthenia and erythematous rash during a stay in Haiti. On his return to Italy, ZIKV RNA was detected in his urine and saliva 91 days after symptom onset, and in his semen on day 188, six months after symptom onset. Our findings support the possibility of sexual transmission of ZIKV and highlight the importance of continuing to investigate non-vector-borne ZIKV infection.

## Case description and laboratory investigations

In the second half of January 2016, a previously healthy man in his early 30s reported to the National Institute of Infectious Diseases in Rome, Italy, a history of five-day self-limiting febrile syndrome (< 38 °C) associated with asthenia and an erythematous rash during a stay in Haiti from mid-January to early February 2016. Zika virus (ZIKV) infection was diagnosed in Haiti by ZIKV-specific IgM serology four days after symptom onset (Figure). He returned to Italy 14 days after symptom onset.

**Figure:**
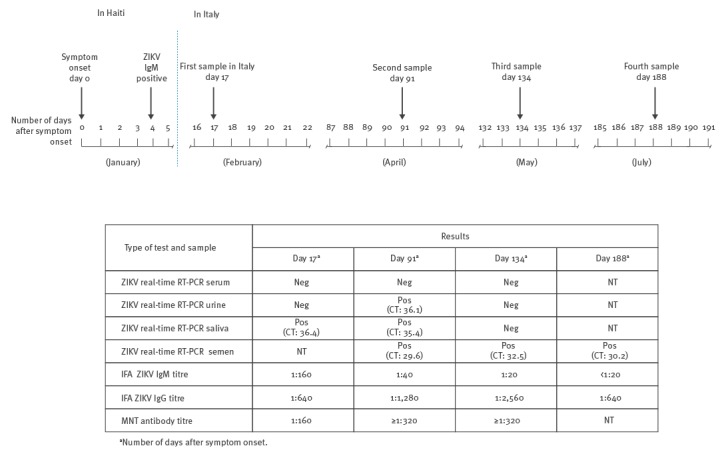
Laboratory findings related to Zika virus infection in a traveller returning from Haiti to Italy, February–July 2016

CT: threshold cycle; IFA: indirect immunofluorescence assay; NT: not tested; Neg: negative; Pos: positive; RT-PCR: reverse transcription-PCR; ZIKV: Zika virus; MNT: microneutralisation test.

Dengue virus and chikungunya virus infections were ruled out following testing of serum and urine samples taken 17 days after symptom onset by both qualitative real-time reverse transcription (RT)-PCR (RealStar Dengue RT-PCR Kit and RealStar Chikungunya RT-PCR Kit, altona Diagnostics, Germany) and serology (indirect immunofluorescence assay (IFA), Arbovirus Fever Mosaic 2, IgM and IgG, Euroimmun, Germany). ZIKV serology (IFA, Arbovirus Fever Mosaic 2, Euroimmun) was positive: ZIKV IgM and IgG antibody titres were 1:160 and 1:640, respectively. Serum ZIKV-specific neutralising antibodies were confirmed by microneutralisation test [1]. ZIKV real-time RT-PCR (RealStar Zika Virus RT-PCR Kit, altona Diagnostics) in saliva was positive with a threshold cycle (CT) value of 36.4; serum and urine samples were both negative. 

Testing of convalescent sera taken 91 and 134 days after symptom onset were ZIKV real-time RT-PCR negative. On day 91, the test was positive for urine, saliva and semen samples, with CT values of 36.1, 35.4, and 29.6, respectively. On day 134, only a semen sample was positive (CT: 32.5). At the subsequent follow-up, on day 188, a semen sample was again positive (CT: 30.2); the patient is still under evaluation. The patient was not affected by any chronic disease or immunological impairment. 

All samples were tested also using a pan-flavivirus NS5 nested RT-PCR (modified from [2]), followed by sequencing of the amplicons (data not shown) to exclude any sample mismatch. 

On day 91, ZIKV IgM and IgG titres were 1:40 and 1:1,280, and on day 134, 1:20 and 1:2,560, respectively. 

ZIKV isolation on Vero-E6 cells was attempted with all the collected samples. Briefly, bodily samples were diluted 1:5 in serum-free Dulbecco’s-modified Eagle’s medium (D-MEM) with antibiotics, inoculated into Vero-E6 cells that were 24 hours-old and then incubated for 60 minutes at 37 °C. After incubation, D-MEM with 2% heat-inactivated fetal bovine serum was added. The cells were followed daily for the appearance of cytopathic effects. After seven days, the cells were subcultured by scraping them and adding fresh cells. Each blind subpassage (three times) was checked for the presence of ZIKV RNA by real-time RT-PCR. No ZIKV isolates were obtained from samples collected during the convalescent phase.

Throughout the course of the ZIKV infection, the patient always had protected sexual intercourse with his spouse, using condoms. His spouse did not report ZIKV-related symptoms, and as at 18 July 2016, her ZIKV serology was still negative. 

## Background

Zika virus is a single-stranded RNA virus (genus *Flavivirus*) mainly transmitted by the *Aedes* mosquito, as well as through sexual contact with symptomatic and, possibly, asymptomatic individuals [3,4]. This non-vector-related mode of transmission was first described in 2008 in the United States [5] and was then reported in several other countries [3,4,6,7].

ZIKV RNA can be detected in different bodily fluids with a wide range of viral loads, depending on the sampling time since acute infection [8,9]. ZIKV from human semen samples has been isolated in African green monkey Vero cells [10] and higher viral loads have been detected in sperm compared with other bodily samples during the convalescent phase [11]. Previous reports have shown that ZIKV RNA has been detected in semen up to day 62 after symptom onset [12-14]. Taken together, these data suggest that virus could replicate specifically in the male genital tract and may persist in semen, with implications for potential male-to-female sexual transmission, even in the absence of haematospermia.

## Discussion

In previous reports, convalescent phase saliva and urine samples were positive by ZIKV real-time RT-PCR in 39 days after symptom onset [3,14].

For the case described here, detection of ZIKV RNA in urine and saliva 91 days after symptom onset and in semen up to day 134 might indicate a possible role played by other non-vector modes of transmission during kissing or vaginal, oral and anal sex. Because of the lack of virus isolation from all the collected samples, we cannot definitively state that saliva, urine and semen represent a potential source of ZIKV that could be transmitted without a vector. During the outbreak in French Polynesia, ZIKV was more frequently detected in saliva than in blood after the first week from symptom onset [13] and it was isolated on day 6 from the saliva of a patient during acute ZIKV infection [14]. No cases involving ZIKV transmission through biological fluids other than semen have been reported, but potential transmission of ZIKV through saliva warrants investigation [15].

The detection of ZIKV RNA in semen up to day 134 might indicate a prolonged potential risk for sexual transmission, for a period longer than previously reported [12]. In reports of Ebola virus disease, suspected sexual transmission of Ebola virus occurred 179 days after onset of the disease [16] and Ebola virus RNA has been detected in semen for 4–6 months after disease onset in 43% of survivors [17].

The lack of isolation of ZIKV from the various biological samples of our patient, during the convalescent phase, is not unexpected. The high CT values found are consistent with a low Zika viral load during the convalescent phase of infection, making it difficult to obtain viral cultures and thus sequence data. 

Because of prolonged detection of ZIKV RNA and isolation of replication-competent virus in semen [11,13], the testes are considered an immunoprivileged replication site for ZIKV [18]. Seminal shedding of ZIKV seems to coincide with the duration of spermatogenesis (69–80 days), suggesting a hypothesis of infection of sperm progenitors and viral shedding during the differentiation process [18]. Our results showed the persistence of ZIKV RNA for 188 days after symptom onset, but this is not sufficient to support a hypothesis of ZIKV RNA being present in sperm progenitors until spermatozoa are fully differentiated and eliminated. Further studies are needed in order to understand persistence of ZIKV in semen and the potential risk of ZIKV sexual transmission.

### Public health impact

The European Centre for Disease Prevention and Control and the World Health Organization recommend that all travellers returning from areas with ongoing ZIKV transmission should adopt safer sex practices or consider abstinence for at least eight weeks after their return [4,19]; if men have ZIKV-related symptoms, they should adopt safer sex practices or consider abstinence for at least six months.

Considering the 80% incidence rate of asymptomatic ZIKV infection [20], further studies are needed to assess viral persistence in asymptomatic men and the potential risk for sexual transmission and fetal abnormalities following infection during pregnancy. The prolonged genital shedding reported here may have implications for screening measures to detect ZIKV RNA for semen cryopreservation in sperm banks [21].
